# Ultra-broadband Tunable Resonant Light Trapping in a Two-dimensional Randomly Microstructured Plasmonic-photonic Absorber

**DOI:** 10.1038/srep43803

**Published:** 2017-03-03

**Authors:** Zhengqi Liu, Long Liu, Haiyang Lu, Peng Zhan, Wei Du, Mingjie Wan, Zhenlin Wang

**Affiliations:** 1 School of Physics and National Laboratory of Solid State Microstructures, Nanjing University, Nanjing 210093, China.; 2 College of Physics and Communication Electronics, Jiangxi Normal University, Nanchang 330022, China; 3 Collaborative Innovation Center of Advanced Microstructures, Nanjing 210093, China

## Abstract

Recently, techniques involving random patterns have made it possible to control the light trapping of microstructures over broad spectral and angular ranges, which provides a powerful approach for photon management in energy efficiency technologies. Here, we demonstrate a simple method to create a wideband near-unity light absorber by introducing a dense and random pattern of metal-capped monodispersed dielectric microspheres onto an opaque metal film; the absorber works due to the excitation of multiple optical and plasmonic resonant modes. To further expand the absorption bandwidth, two different-sized metal-capped dielectric microspheres were integrated into a densely packed monolayer on a metal back-reflector. This proposed ultra-broadband plasmonic-photonic super absorber demonstrates desirable optical trapping in dielectric region and slight dispersion over a large incident angle range. Without any effort to strictly control the spatial arrangement of the resonant elements, our absorber, which is based on a simple self-assembly process, has the critical merits of high reproducibility and scalability and represents a viable strategy for efficient energy technologies.

Light-trapping structures hold great promise for a variety of applications, such as photodetectors and photovoltaic devices^1^,^2^,^3^,^4^. Conventional techniques, such as surface texturing^5^,^6^ and interference-based antireflection coating (ARC)^7^,^8^, could be applied to realize efficient light trapping by inhibiting light from reflecting directly back into the incident space and increasing the optical path length in the photoactive materials. To address the two most important aspects of light trapping, which are the in-coupling of light and the density of optical states in the photoactive materials, properly designed nanophotonic light-trapping microstructures have recently been proposed to allow for more energy to be coupled into the active media of a photoelectric converter and to increase the light-matter interaction. High-index nanoscale wires^9^, particles^10^,^11^, and shells^12^ could support strong optical resonances owing to the excitations of optical modes such as Mie modes, whispering gallery modes and guided-like modes, which have provided ideal building blocks for versatile light-trapping layers or super-absorbing films^4^.

On the other hand, based on their strong light concentration and scattering properties, light-trapping layers employing metallic plasmonic microstructures have gained significant attention recently^1^,^2^,^13^,^14^. Efficient resonant light absorption by various plasmonic nanostructures, including microcavities^15^, gratings^16^, and gap-plasmon resonators^17^,^18^, has been widely studied. Since the first demonstration by Landy *et al*.^19^, metamaterial absorbers, typically composed of a dielectric thin film sandwiched between an opaque conducting back plate and a lithographically patterned metallic microstructure, are recognized as promising candidates to realize near-unity electromagnetic wave absorption at microwave^20^, terahertz^21^ and infrared-visible frequencies^22^,^23^,^24^, and extensive follow-up work has been carried out to make the absorbers wide-angle incidence compatible. However, because the electric and magnetic resonance frequencies of metamaterials are determined by their specific geometries, the absorbers based on them generally exhibit narrow absorption bandwidths, which severely limits their applications in light energy harvesting and photoelectric conversion. One of the most popular and effective ways to extend the working bandwidths of the absorbers is by blending dimensionally dispersed metallic resonators and integrating the corresponding resonances into a broad unity absorption band^25^,^26^. To realize a relatively broad and spectrally flat high absorption band, gradually varied metallic resonators with almost continuously changing in dimension like a trapezoidal^27^ or saw-tooth^28^,^29^,^30^ geometry have been proposed and demonstrated. Nevertheless, their complex and elaborate nanofabrication requirements make them inherently difficult to produce over large areas and hence limit their applications.

In addition to using deterministic nanophotonic architectures, structural disorder can offer an alternative strategy to improve light-trapping efficiency over a broad spectral range by light diffusion, multiple-scattering, and light coupling among the neighboring scatterers^31^. The introduction of structural randomness, including random spatial positions^32^ and sizes^33^,^34^ of the scatterers, has been studied to realize optical management with broad spectral and angular responses. For instance, a thin photoactive film drilled with a random pattern of holes demonstrates greater wideband light-trapping than one without holes and particularly greater light-trapping than a periodic holes array; this might provide a powerful approach for photon management in energy efficiency technology, which could benefit from a lower amount of materials used and the possibility of better photoelectron conversion^35^. Regarding metal-based absorbers, broadband absorption enhancement led by randomly patterned or stacked plasmonic resonators has also been presented^36^,^37^. More importantly, compared to elaborate periodic microstructures, random microstructures usually require relatively simple and low-cost fabrication methodologies, and their optical properties are expected to be less susceptible to imperfections.

Colloidal microspheres and their corresponding arrays are effective platforms to realize light-trapping by excitations of the localized resonances and coupled guided modes^38^,^39^,^40^. Prepared through two-dimensional (2D) colloidal crystal templating, spherical micro-void arrays buried in metal show omnidirectional high absorption that relies on the excitation of localized surface plasmon resonances^41^,^42^. Furthermore, taking advantage of low quality whispering gallery modes supported by size-dispersed colloidal nanoshells, broadband absorption enhancement could be achieved^12^. In this letter, we propose and demonstrate a novel 2D hybrid plasmonic-photonic absorber by randomly patterning a monolayer of monodispersed gold-capped polystyrene (PS) microspheres on a flat, optically opaque gold film. Compared to its periodic counterpart, our proposed absorber displays a broad near-unity absorption band due to the excitation of multiple hybrid plasmonic-photonic modes^43^,^44^,^45^,^46^,^47^ and is scalable by tuning the size of the PS microspheres. In order to expand the absorption bandwidth, two different-sized gold-capped PS microspheres are mixed into a densely packed monolayer on a gold back-reflector. As a proof-of-concept investigation, two types of PS microspheres with diameters of 1.019 μm and 1.587 μm are selected to fabricate the absorber in the near-infrared regime through a simple self-assembly approach. By optimizing the structural parameters of the absorber, such as the mixture ratio of these two different-sized microspheres and the thickness of the metallic cap over the PS beads, an ultra-broadband infrared absorption arising from effective modes blending and coupling effects is identified that shows desirable optical trapping in dielectric region and slight dispersion over a wide incident angle range while maintaining an average absorption greater than 90%. In principle, this absorption band can be easily scaled over a wide frequency range by tuning the PS colloids sizes. This work offers a facile and cost-effective strategy to fabricate an ultra-broadband perfect absorber with a very large area, and it demonstrates great potential for applications in optoelectronic devices based on high-efficiency light-harvesting.

## Results

### A randomly microstructured plasmonic-photonic absorber

**Figure 1:**
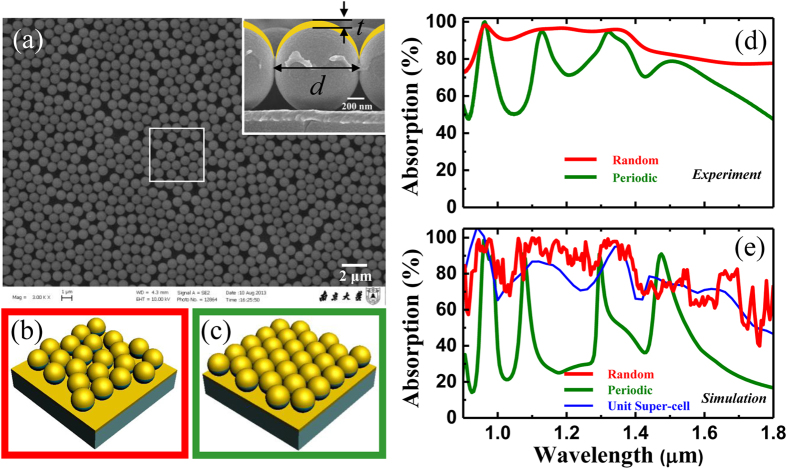
A broadband photonic-plasmonic absorber and its optical absorption. (**a**) SEM image (scale bar: 2 μm) of a randomly patterned gold-capped PS microsphere monolayer on a 150 nm-thick gold back-reflector. Here, the diameter (*d*) of the PS microsphere is 1.019 μm and the nominal thickness (*t*) of the gold cap is 20 nm. The inset is a cross-sectional SEM image of an individual element. Schematic of a unit super-cell in the randomly organized microstructure (**b**) from the digitized SEM image framed by the white box in a) and that of the periodic gold-capped PS microsphere array (**c**). Measured (**d**) and calculated (**e**) absorption spectra for a random plasmonic-photonic absorber (red curve) and for a periodic absorber (green curve). The blue curve in (**e**) is the calculated absorption of the unit super-cell shown in (**b**) using scattering boundary conditions.

The plasmonic-photonic microstructures were prepared by self-assembling a densely packed monolayer of monodispersed PS microspheres onto a quartz substrate pre-coated with an optically opaque, 150 nm-thick gold film, followed by the deposition of a 20 nm-thick (*t*) semi-shell on top of each colloid (details shown in the Experimental section and Supplementary Fig. S1). In order to obtain disordered microstructures with a large area (over 1 × 1 cm), a well-developed self-assembly method^48^, which controlled the evaporation rate of the suspension (comparatively rapid evaporation rate) under ultrasonic disturbance, was utilized. Figure 1(a) shows a top-view scanning electron microscope (SEM) image of the resultant monolayer, which is randomly patterned by gold-capped PS colloids with a diameter (*d*) of 1.019 ± 0.02 μm. The monolayer apparently possesses a weak statistical short-range correlation in the position of colloids but lacks any long-range order, and the inset shows a magnified cross-sectional SEM image of an individual gold-capped PS microsphere on a gold back-reflector.

Reflection (*R*) and transmission (*T*) spectra were measured using a commercial Fourier transform infrared (FTIR) spectrometer (Nicolet-5700). Due to the presence of an optically opaque gold back-reflector, the transmission of the sample vanishes, and thus only reflection measurements are required to deduce the light spectral absorption, (*A*), by *A*(*λ*) = 1 − *T*(*λ*) − *R*(*λ*) = 1 − *R*(*λ*). Due to the restriction of a customized experimental setup, reflectance measurements were performed at a small incident angle (~8°) and were considered to be a good approximation of light reflectance under normal incidences of light. Figure 1(d) demonstrates the measured absorption spectrum of the as-prepared microstructure with the randomly patterned monolayer of 1.019 μm gold-capped PS colloids (red curve) and indicates an excellent absorption performance covering a broad spectral range from ~0.9 μm to ~1.3 μm, with average absorptivity higher than 90%. To illustrate the possibility that the introduction of a random colloid pattern offers broadband photon management, the absorption measurement for a gold-capped PS colloid monolayer of a perfectly hexagonally close-packed (HCP) array on gold film was also performed and is shown as the green line in Fig. 1(d) (SEM image of the sample shown in Supplementary Fig. S1), and it is clear that the integrated absorption exhibits several sharp absorption peaks, which is quite different from that of the random structure.

To compare the absorption performance of the random microstructure with its periodic counterpart, three-dimensional full-field electromagnetic simulations under normal incidence were conducted using a commercial finite-element-method-based software package (COMSOL Multiphysics), and the corresponding calculated absorption spectra normalized to the intensity of the incident light are plotted in Fig. 1(e) for the random (red curve) and ordered (green curve) gold-capped PS colloidal monolayers. In the simulations, the modeling geometry parameters are the same as those of the experiments, and to mimic the infinitely large area of the sample, periodic boundary conditions were used (see Method section). In particular, Fig. 1(b) depicts the schematic representation of a unit super-cell consisting of more than twenty randomly organized gold-capped PS microspheres for numerical simulation, which was obtained from the digitized SEM image outlined by the white box in Fig. 1(a). And the spectral absorption for this unit cell is shown as blue curve in Fig. 1(e) by utilizing the scattering boundary conditions. Overall, the comparative results show good agreement between the simulated and the measured absorption spectra.

### Elucidation of absorption broadening effect of the disorder microstructure

**Figure 2:**
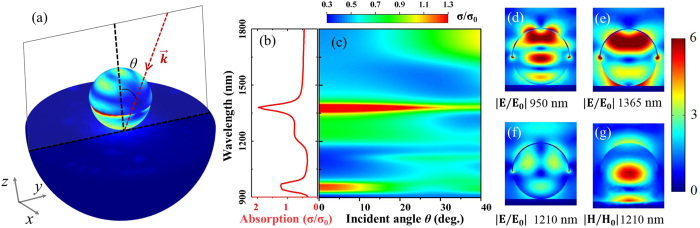
Calculated absorption properties of a single gold-capped PS microsphere on a metal back-reflector. (**a**) Schematic of the calculation model for a single gold-capped PS microsphere on gold back-reflector with the same geometrical parameters as above. The magnetic field (*H*) of the incident plane wave is perpendicular to the incident plane (*yoz*), which represents TM incidence. (**b**) The simulated absorption cross-section (*σ*/*σ*_0_) as a function of the incidence wavelength under normal incidence, and (**c**) the incident angle (*θ*) dependence of the absorption cross-section for this single resonator. (**d**–**g**) The calculated electric/magnetic field distributions for the cross-section of the gold-capped microsphere (along the *yoz* plane) at wavelengths *λ*_1_ = 0.950 μm, *λ*_2_ = 1.210 μm, and *λ*_3_ = 1.365 μm at normal incidence, respectively.

The metal-capped microspheres could support a series of cavity-like resonances, where the electromagnetic field had a nondispersive localization^49^ within the spatial region between the metallic back plate and the top semi-shells. To estimate the efficiency of light trapping, numerical simulations with top-illumination over a single 1.019 μm PS microsphere capped by a 20 nm-thick gold semi-shell on a gold back-reflector were performed under scattering boundary conditions, as in the schematic in Fig. 2(a). Figure 2(b) illustrates the absorption cross-section (*σ*) defined by the ratio of the absorbed energy to that of the incidence as a function of illumination wavelength, which was normalized to the geometrical cross-section (*σ*_0_) of the gold-capped PS microsphere. Three resonant absorption peaks located at *λ*_1_* = *1.365 μm, *λ*_2_* = *1.210 μm, and *λ*_3_* = *0.950 μm were clearly observed, which demonstrated very weak dispersion over the large range of incident angles, as shown in Fig. 2(c). The nature of these three distinct optical modes could be identified by the electric/magnetic field profiles of a single resonator in Fig. 2(d–g). According to the electric field distributions presented in Fig. 2(d and e), the absorption peaks centered at *λ*_1_* = *1.365 μm and *λ*_3_* = *0.950 μm could be regarded as plasmonic-coupled Fabry-Perot (FP)-like resonances supported within the region between the top gold semi-shells and the gold back-reflector with low quality factors^50^. For the absorption peak centered at *λ*_2_* = *1.210 μm, as shown in Fig. 2(f), most of the electrical field was trapped in the PS microspheres and the interstice between the PS colloids and the bottom gold plate, which corresponds to the excitation of the localized surface plasmons and the (lowest transverse) magnetic plasmon cavity resonances identified by the magnetic field distribution shown in Fig. 2(g). Such electric and magnetic field distributions imply that the optical field was mostly confined within the region occupied by the lossless dielectric PS colloids, confirming the strong light confinement capabilities of our proposed microstructure for photon management in energy efficiency.

By patterning these individual gold-capped PS microspheres in a dense ensemble with an HCP lattice on the thick gold back-reflector, the topology of this periodic microstructure allows for effective diffractive coupling of incident light, routing it into the in-plane propagation modes. It is worthwhile to note that a ~20 nm-thick gold semi-shell could lead to an electrically connected corrugated metal film on the surface of the 2D HCP PS microsphere array and consequently form a quasi-3D plasmonic-photonic crystal^51^,^52^. As such, the domination over the features of the sharp absorption peaks, as shown in the green curves in Fig. 1(d and e), was actually the consequence of the excitation of coupled Bloch eigenmodes, which are either guided in the 2D PS microspheres array slab due to the refractive index contrast or are bound at the interface of the gold semi-shell arrays and the gold bottom film as propagating surface plasmons modes^49^,^53^,^54^ (see corresponding electric field distributions in Supplementary Fig. S2). When the gold-capped PS microsphere monolayer is randomly distributed over the gold reflector, these Bloch eigenmodes are suppressed by the lattice disorders. In this case, for an ensemble of microcavities, resonances of an individual hybrid cavity must be strongly influenced by electromagnetic interactions, including near-field coupling of nearly physically touching cavities, due to the nature of the leakage of the cavity resonances and far-field interactions mediated by the scattered light field of the individual resonator, which eventually leads to a spectral feature with relatively broad bandwidth, shown as the red curves of the measured and simulated results in Fig. 1(d and e).

**Figure 3:**
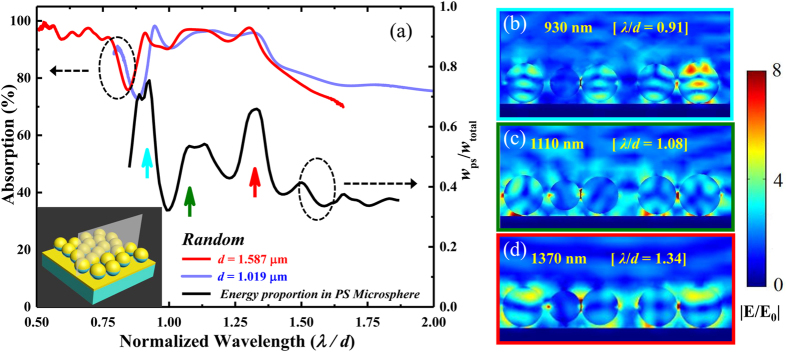
Scalability of the absorption of the randomly microstructured plasmonic-photonic absorber. (**a**) The measured absorption spectra of a randomly microstructured plasmonic-photonic absorber made of 1.587 μm (red curve) or 1.019 μm (purple curve) diameter PS colloids as a function of normalized wavelength (*λ/d*), and the numerically estimated spectral energy density in the PS microsphere (black curve). (**b**–**d**) The calculated electric field distributions along the grey plane of the schematic super-cell of the absorber (inset in (**a**)) for three typical absorption modes at normalized wavelengths of 0.91, 1.08, and 1.34, respectively. All the color field maps use the same linear scale.

By tuning the structural parameters of our proposed plasmonic-coupled microcavities, the spectral response can be calibrated to a desired wavelength band, and the optical absorption efficiency can also be optimized accordingly. In Fig. 3(a), the measured absorption spectra are plotted for the random hybrid plasmonic-photonic microstructures made up of 1.019 μm and 1.587 μm PS microspheres with a 20 nm-thick gold-cap coating. The absorption curves are normalized to the diameters of the PS microspheres and indicate that the wavelength of the absorption band can be effectively tuned by changing the diameters of the PS microspheres, regardless of the materials dispersion of the gold. On the other hand, the varying nominal thickness of the gold on each PS microsphere will also have a pronounced effect on the optical properties (see Supplementary Fig. S3), which might be the major cause of the small shift in the normalized absorption spectra of samples with different-sized PS microspheres despite the gold cap being the same thickness (*t*). In addition, for the random hybrid plasmonic-photonic absorber, the numerically estimated energy trapped in PS microspheres (*w*_*ps*_) compared with that trapped in the whole microstructure (*w*_*total*_) as a function of normalized wavelength is shown as a black line in Fig. 3(a). Constructing maps of electric field distributions can provide more insight into the nature of the absorption processes of this random microstructure. As shown in Fig. 3(b–d), the simulated electric field distributions along the grey plane of the schematic super-cell of our absorber (inset in Fig. 3(a)) are plotted for three absorption bands at normalized wavelengths (*λ*/*d*) of 0.91, 1.08, and 1.34. Clearly, a considerable amount of energy is trapped in the lossless dielectric microspheres, especially when these resonant absorption modes are excited.

### Ultra-broadband plasmonic-photonic absorber based on the mixture of different-sized micro-resonators

**Figure 4:**
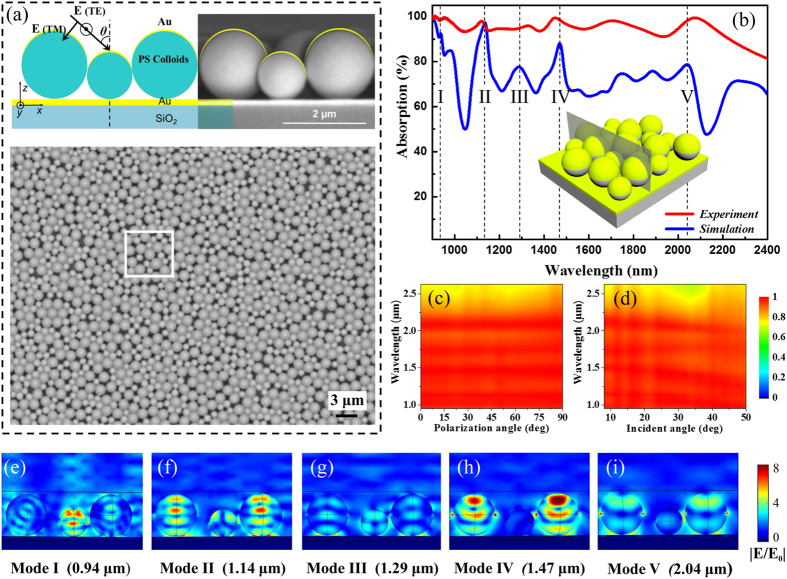
Ultra-broadband plasmonic-photonic absorber based on a mixture of gold-capped PS colloids with diameters of 1.019 μm and 1.587 μm. (**a**) Top-view SEM image of a large-area plasmonic-photonic absorber. The top panel contains schematic and cross-sectional SEM images of the sample, and the white box corresponds to a stochastic region used as a unit super-cell for numerical simulation. (**b**) Measured light absorption spectrum of the absorber under an incident angle of 8° and the simulated absorption spectrum under normal incidence. (**c**) Measured absorption spectra of the hybrid plasmonic-photonic microstructure as a function of the polarization angle with 8° incident angle. (**d**) Incident angle dependence of absorption spectra. (**e**–**i**) The calculated electric field distributions of five absorption bands centered at the wavelengths marked in (**b**).

So far, we have demonstrated that the random microstructure, which possesses a weak spatial correlation of the position of the metal-capped PS microspheres, can result in a broadband optical response instead of exhibiting sharp spectral features. From the point of sample preparation, however, the monodispersed microspheres prefer to self-assemble via an entropy-driven process into an ordered array with the HCP lattice. Mixing colloidal suspensions of different-sized PS microspheres is an effective way to introduce a certain type of disorder into the self-assembly process. Figure 4(a) demonstrates the SEM image of a resultant random gold-capped colloid monolayer on a gold back-reflector prepared by mixing equal volumes of aqueous suspensions of PS colloids with diameters of 1.019 μm and 1.587 μm, which have the same solid concentration of 1 wt. %. More importantly, broadening of the absorption band is expected because of the blending of the optical resonant modes arising from the different-sized micro-resonators. As shown in the red curve in Fig. 4(b), the plasmonic-photonic absorber composed of the two sizes of PS microsphere presents a measured ultra-broad absorption band from approximately 0.9 μm to 2.2 μm with average absorptivity higher than 90%. The numerical simulation, shown as the blue curve, was performed using periodic boundary conditions on a relatively large complex unit super-cell, which was obtained from a digitized SEM image stochastically framed by the white box in Fig. 4(a). Although the measured spectrum looks smoother and with higher absorptivity because the simulation model is somewhat inconsistent with the real random, dense microstructure, the simulated absorption spectrum almost faithfully reproduces the main features of the measured spectrum. Analysis of the electric field distribution suggests that the ultra-broadband unity absorption of our microstructure originates from blending and coupling effects of the resonant modes supported by the neighboring different-sized gold-capped PS microspheres. Along the vertical cross-section marked in the inset of Fig. 4(b), the simulated electric field distributions specific to five typical absorption peaks at the wavelengths of (i) 0.94 μm, (ii) 1.14 μm, (iii) 1.29 μm, (iv) 1.47 μm, and (v) 2.04 μm are summarized in Fig. 4(e–i). Each absorption peak corresponds to the excitation of localized optical modes confined by different-sized resonators, such as the plasmonic-coupled FP-like modes and the magnetic plasmon cavity modes, as well as the corresponding couplings. Clearly, some absorption peaks (marked as (i)-(iii) and (v)) are the contributions of the resonant modes excited coincidently in both different-sized resonators. The others, like absorption peak (iv), are attributed to the optical excitation of only one resonator, while the other resonator is off resonance. It follows that the total absorption bandwidth can be effectively widened by properly selecting the diameters of the dielectric colloidal microspheres, and in principle, this ultra-broadband light trapping could be easily scaled down to the visible regime by utilizing the monolayer of mixed gold-capped colloids with combinations of two small, different-sized PS microspheres with suitable particle size ratios and optimized nominal gold-cap thickness (see Supplementary Fig. S3). Due to the highly symmetric configuration of the microsphere itself^41^ and the randomness of the structure, the resonant broadband absorption is expected to be polarization-independent under near-normal incidence, which is illustrated by the measured absorption spectra as a function of both polarization angle and wavelength in Fig. 4(c). We conducted additional studies to investigate the incident angle dependence of our proposed ultra-broadband light absorber. The absorption spectra with different incident angles (*θ*, as shown in Fig. 1(c)) have been experimentally studied, as shown in Fig. 4(d), and overall, the ultra-broadband absorption with high average absorptivity (above 90%) is maintained even at large incident angles of up to 50°. This is also a significant achievement for our random microstructure absorber, owing to the nature of the localized resonances and the corresponding coupled modes excited in the plasmonic-photonic microstructure^39^,^40^.

**Figure 5:**
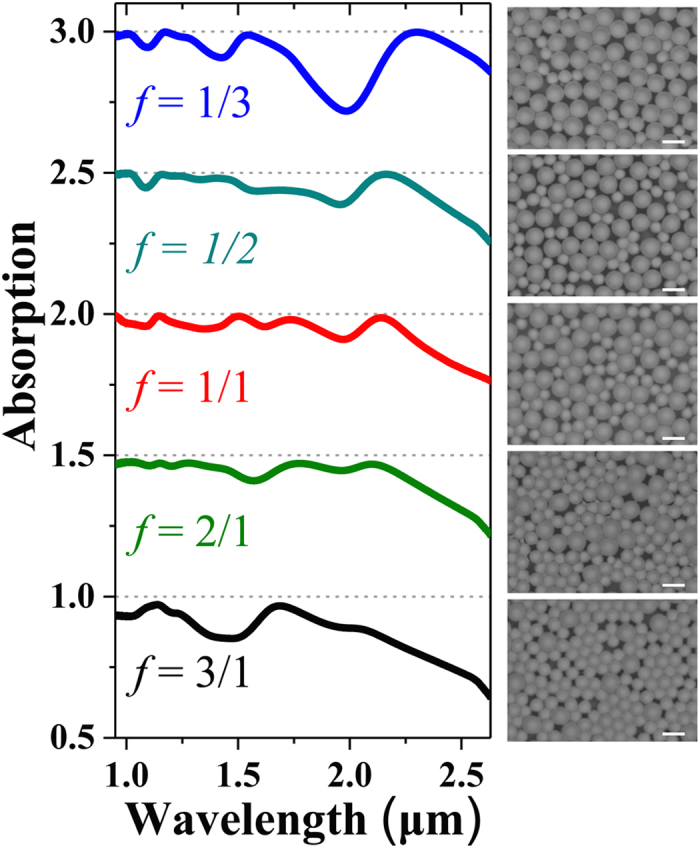
The spectral absorption evolution of the disordered microstructures in the volume mixture ratio (*f = V*_*small_PS*_: *V*_*large_PS*_) of colloidal suspensions containing PS microspheres of 1.019 μm and 1.587 μm in diameter. Left column: *f* is increased from 1/3 to 1/2, 1/1, 2/1, and 3/1. The nominal Au layer thickness is kept at *t* = 20 nm, and the spectra are sequentially offset by 0.5 for clarity. Right column: the corresponding SEM images of the 2D plasmonic-photonic microstructure corresponding to different values of *f*. All scale bars represent 2 μm.

In addition, we studied the evolution of the absorption spectrum by changing the mixture ratio (*f*), which was defined as the mixture volume ratio of the 1.019 μm-diameter PS colloid suspension (*V*_*small_PS*_, 1 wt. %) to the 1.587 μm-diameter PS colloid suspension (*V*_*large_PS*_, 1 wt. %). When *f* was increased from 1/3 to 1/2, 1/1, 2/1 and 3/1, modification of the absorption spectrum is clear, as shown in Fig. 5. For instance, for the as-prepared sample with *f* = 1/3 (or 3/1), the shape of the absorption spectrum is very similar to that of the random single-sized absorber made of 1.578 μm (or 1.019 μm) PS microspheres only; this is mainly the result of the PS microsphere with the dominant quantity in this plasmonic-photonic absorber with two sizes of PS microspheres (as the SEM images show in the right column of Fig. 5). A flat and ultra-broad high absorption band could be achieved when *f* was optimized to 1/1. However, taking the cases of *f* = 1/2 and 2/1 into account, the absorption spectra only show minor variations in the spectral line shape in the case of *f* = 1/1, which implies that the ultra-broadband light absorption could be obtained in an appropriate range of fluctuant mixture ratios. This feature indicated an excellent fault tolerance for the fabrication of our proposed random plasmonic-photonic microstructure to realize an ultra-broadband high optical absorption for practical applications.

## Conclusion

In summary, we demonstrated a novel 2D hybrid plasmonic-photonic absorber by randomly patterning a monolayer of single-sized metal-capped dielectric microspheres on a metal film. The experiments and numerical simulations show that the random spatial arrangement of the metal-capped microspheres greatly broadened the absorption band. The dependence of the resonance on the geometries of the microstructure provides freedom in the design of specific wavelengths and improving the absorption efficiency. The wideband near-unity absorption mechanism can be inferred by the simulated field distributions, in which it is very clear that a much stronger photonic density of states is confined in the lossless dielectric region. To expand the absorption bandwidth further, two different-sized metal-capped dielectric microspheres were mixed into a densely packed monolayer on metal film. By properly selecting the sizes of the microspheres and optimizing their mixture ratio, an ultra-broadband near-unity absorption for the target wavelength was achieved, owing to the effective mode blending and coupling effects. This ultra-broad absorption band demonstrated a perfect desirable optical trapping in dielectric region and slight dispersion for incident angles over a wide angular range of 50° while maintaining an average measured absorption greater than 90%. This work provides a facile and cost-effective strategy to fabricate an ultra-broadband hybrid plasmonic-photonic perfect absorber composed of a very large area of complex non-periodic array of scatterers. Its merits include simple technical requirements, low-cost, large area, high reproducibility, and easy scalability, making the ultra-broadband light absorber demonstrated here an outstanding candidate for applications in energy harvesting, biology and optical storage.

## Methods

### Fabrication of samples

Fabrication of hybrid plasmonic-photonic microstructures began by evaporating a 150 nm gold layer onto a clean quartz substrate. An ion-beam coater (IBC Model 682, Gatan Corp.) was used to deposit the gold layer with the desired thickness under a vacuum of 5 × 10^−6^ Torr (1 Torr ≈ 133 Pa) at a rate of 0.66 Ås^−1^. The obtained quartz glass coated with the gold layer was soaked in a 20 mM 3-mercapto-1-propanesulfonic acid, sodium salt HS-(CH_2_)_3_-SO_3_Na water solution overnight, forming a monolayer of hydrophilic molecules on the gold surface^46^. Aqueous suspensions of the colloid mixtures with different volume ratios were then injected into a cell formed by sandwiching a U-shaped enamel-wire spacer (1 mm in diameter) between the gold-coated quartz substrate and another clean quartz slide. All the PS colloids used in our experiments were purchased from Duke Corp. After drying in air (controlling the evaporation rate of the suspension under ultrasonic disturbance) at room temperature, densely packed colloid monolayers were grown on the metal-coated quartz substrate based on to capillary force of the cell^48^. Finally, thin gold layers were sputtered onto the microsphere monolayers. For comparison, we also fabricated the periodic plasmonic-photonic microstructures that were composed of 2D colloidal crystals with a perfect HCP lattice using our well-developed self-assembly method^40^.

### Optical measurements

The reflectivity and transmission were measured using a customized FTIR spectrometer (Nicolet 5700). For oblique incidence measurements, the samples were mounted on a home-made variable-angle reflection accessory to collect the angle-resolved reflectivity in 2° increments to a maximum incident angle of 50°. In each case, the reflectivity was determined by comparing the measured values to the reflectivity of a gold mirror, which acted as a reference. The optical spot size of the incident beam on the sample was approximately 0.8 mm. Polarization-dependent measurements were performed with an adjustable polarizer. Sample structures were characterized by SEM (FEI Philips XL-30).

### Numerical simulations

Three-dimensional numerical simulations were performed using a commercial software package (COMSOL Multiphysics) based on the finite-element method. The maximum mesh sizes of the gold layer, PS, and air were set to 13.5 nm, 90 nm and 150 nm, respectively. The periodic boundary conditions were adopted in the x and y directions for the periodic metal-capped PS microsphere simulations. A perfectly matched layer condition was imposed at the boundaries along the z-axis. The relative permittivity of gold was described by the Drude mode, 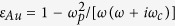, with the plasma frequency, *ω*_p_ = 8.99 eV, and the collision frequency, *ω*_c_ = 0.0269 eV^43^,^45^. The refractive index of the PS was taken as 1.59^45^. The absorption (*A*) was deduced by recording the reflected and transmitted fluxes, *R* and *T*. Because of the presence of the optically opaque gold back-reflector, there was no light transmitted through the sample, and thus the absorption satisfies the relation *A = *1 *− T − R = *1 *− R.* The proper energy density that accounted for the energy stored in the non-magnetic materials where the permittivity was dispersive and absorptive could be derived by Ruppin’s formalism, 
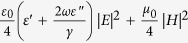
, where ε′ and ε″ represent the real and imaginary parts of the dielectric permittivity of the materials and *γ* is the damping frequency^55^.

## Additional Information

**How to cite this article:** Liu, Z. *et al*. Ultra-broadband Tunable Resonant Light Trapping in a Two-dimensional Randomly Microstructured Plasmonic-photonic Absorber. *Sci. Rep.* **7**, 43803; doi: 10.1038/srep43803 (2017).

**Publisher's note:** Springer Nature remains neutral with regard to jurisdictional claims in published maps and institutional affiliations.

## Supplementary Material

Supplementary Information
