# Supplementary material for: Ultra-broadband Tunable Resonant Light Trapping in a Two-dimensional Randomly Microstructured Plasmonic-photonic Absorber
Source: Sci Rep. 2017 Mar 3;7:43803. doi: 10.1038/srep43803 (PMC5335598; doi:10.1038/srep43803)
Supplement: Supplementary Information [file srep43803-s1.doc]

**Supplementary Information for**

**Ultra-broadband Tunable Resonant Light Trapping in a Two-dimensional Randomly Microstructured Plasmonic-Photonic Absorber**

Zhengqi Liu^1,3,†^*,* Long Liu^1,2,†^*,* Haiyang Lu^1,2^*,* Peng Zhan^1,2,*^, Wei Du^1,2^, Mingjie Wan^1,2^ and Zhenlin Wang^1,2,*^

^1^School of Physics and National Laboratory of Solid State Microstructures, Nanjing University, Nanjing 210093, China.

^2^Collaborative Innovation Center of Advanced Microstructures, Nanjing 210093, China.

^3^College of Physics and Communication Electronics, Jiangxi Normal University, Nanchang 330022, China.

^†^These authors contributed equally to this work.

*^*^*E-mail: zhanpeng@nju.edu.cn; zlwang@nju.edu.cn.


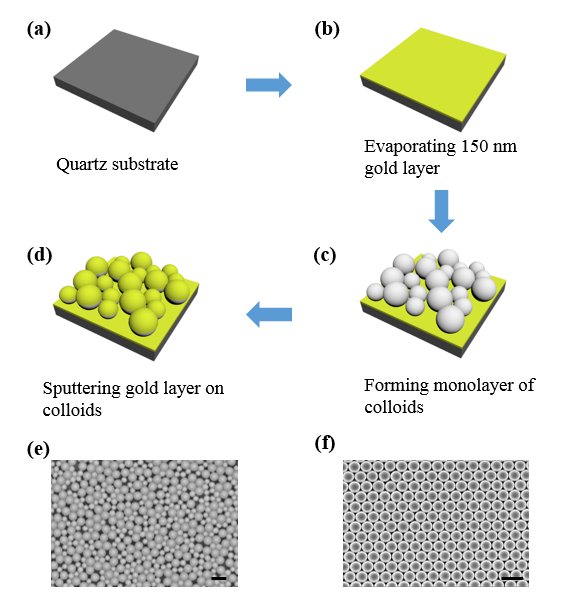


**Supplementary Figure S1.** (**a**)–(**d**) Schematic of the fabrication procedure of the 2D randomly microstructured plasmonic-photonic absorber. The microstructure was prepared by self-assembling a monolayer of PS microspheres on an optically opaque (150 nm-thick) gold film, followed by deposition of a 20 nm-thick gold half-shell on top of each microsphere. Here, the monolayer of randomly patterned PS colloids was prepared by mixing two different-sized PS colloids. (**e**) SEM image (scale bar, 3 µm) of a monolayer of random colloidal crystal generated by mixing gold-capped PS colloids with diameters of 1.019 µm and 1.587 µm using equal suspension volumes with same solid concentrations. (**f**) SEM image (scale bar, 2 µm) of a gold-capped PS colloid monolayer in a perfect hexagonally close-packed (HCP) array on gold film using monodispersed PS microspheres with diameters of 1.019 µm.


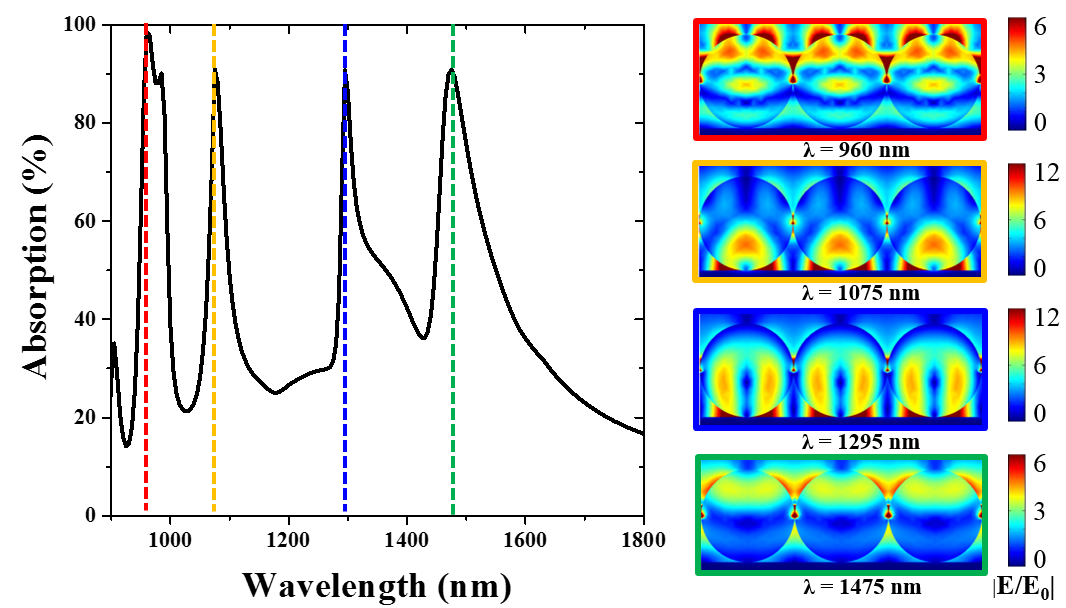


**Supplementary Figure S2.** Calculated absorption spectrum of 2D HCP plasmonic-photonic microstructure consisting of gold-capped PS microspheres with 1.019 µm diameters periodically patterned on 150 nm-thick gold back reflector. The nominal thickness of the gold semi-shell was set as 20 nm. The calculated electric field maps (|***E***|/|***E***_0_|) were plotted at the wavelengths of the four apparently sharp resonant absorption peaks.


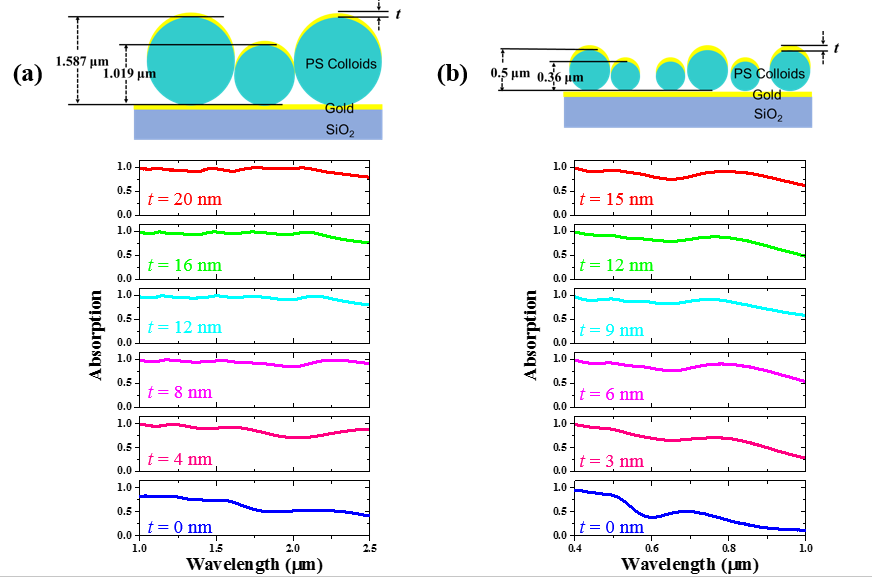


**Supplementary Figure S3.** Measured light absorption spectra dependent on the nominal gold-cap thickness (*t*) for the randomly microstructured plasmonic-photonic absorber consisting of two different-sized gold-capped PS microspheres. Schematic and measured absorption spectra for as-prepared samples made of two different-sized PS microspheres diameters of 1.019 µm and 1.587 µm (**a**), and those with diameters of 0.5 µm and 0.36 µm (**b**).
